# Plastisch-chirurgische Rekonstruktion der unteren Extremität bei alten Patienten

**DOI:** 10.1007/s00113-023-01302-3

**Published:** 2023-03-28

**Authors:** Alexander Haumer, Andreas Gohritz, Martin Clauss, Steven John Lo, Dirk Johannes Schaefer, Rik Osinga

**Affiliations:** 1 Klinik für Plastische, Rekonstruktive, Ästhetische und Handchirurgie, Universitätsspital Basel, Spitalstraße 21, 4031 Basel, Schweiz; 2 Zentrum für Muskuloskelettale Infektionen (ZMSI), Universitätsspital Basel, Spitalstraße 21, 4031 Basel, Schweiz; 3 Klinik für Orthopädie und Traumatologie, Universitätsspital Basel, Spitalstraße 21, 4031 Basel, Schweiz; 4 Canniesburn Plastic Surgery Unit, Glasgow Royal Infirmary, 84 Castle Street, Glasgow, Vereinigtes Königreich; 5 Praxis beim Merian Iselin, Thannerstraße 80, 4054 Basel, Schweiz

**Keywords:** Gebrechlichkeit, Komplexe Wunde, Funktionelle Rekonstruktion, Mikrochirurgie, Rekonstruktive Lappen, Frailty, Complex wound, Functional reconstruction, Microsurgery, Reconstructive flaps

## Abstract

Der Anteil an Patienten jenseits der 7. Lebensdekade in der Bevölkerung steigt weltweit, insbesondere in den hochentwickelten Ländern. Der Bedarf an komplexen Rekonstruktionen der unteren Extremitäten nach Trauma, Tumor oder Infektion in dieser Altersgruppe nimmt zu. Die Rekonstruktion von Weichteildefekten an der unteren Extremität sollte nach dem Prinzip der plastisch-rekonstruktiven Stufenleiter resp. des Aufzugs erfolgen, um schmerzfreies und sicheres Stehen und Gehen zu erhalten. Insbesondere beim älteren Patienten sind sorgfältige multidisziplinäre Planung, gezielte präoperative Beurteilung und Optimierung von Komorbiditäten, wie z. B. Diabetes, Mangelernährung oder pathologischen Gefäßveränderungen, sowie ein altersentsprechendes perioperatives Management erforderlich. Durch die Umsetzung dieser Prinzipien können ältere und hochbetagte Patienten ihre Mobilität und ihre Autonomie, die für eine hohe Lebensqualität entscheidend sind, beibehalten.

**Video online:**

Die Online-Version dieses Beitrags (10.1007/s00113-023-01302-3) enthält 2 Videos.

## Abstract (en)

The proportion of patients in the population beyond the 7th decade of life is increasing worldwide, especially in highly developed countries. Consequently, there is also an increasing need for complex lower extremity reconstructions after trauma, tumors, or infections in this age group. The reconstruction of soft tissue defects of the lower extremity should be performed according to the principle of the plastic-reconstructive ladder or elevator. The goal of reconstruction is to restore anatomy and function of the lower extremity to enable pain-free and stable standing and walking; however, for older patients in particular, a careful preoperative multidisciplinary planning, detailed preoperative assessment and optimization of comorbidities, such as diabetes, malnutrition or pathological vascular alterations, as well an age-adapted perioperative management are necessary. By implementing these principles, older and very old patients can maintain their mobility and autonomy, which are crucial for a high quality of life.

## Lernziele

Nach der Lektüre dieses Beitrags

- können Sie für geriatrische Patienten einschätzen, wann orthoplastisch-rekonstruktive Maßnahmen der unteren Extremität sinnvoll sind.
- können Sie die orthoplastischen Prinzipien und besonderen Strategien, die beachtet werden müssen, verstehen.
- erkennen Sie, weshalb eine enge multidisziplinäre Betreuung dieser Patienten unabdingbar ist.
- gelingt Ihnen die wichtige Unterscheidung zwischen Alter und Gebrechlichkeit.
- können Sie die relevanten präoperativen Abklärungen, die für diese Rekonstruktionen notwendig sind, benennen.
- erfassen Sie, unter welchen Umständen auch im hohen Alter eine freie Lappenplastik als plastisch-rekonstruktive Maßnahme sinnvoll ist.
- kennen Sie die für geriatrische Patienten wichtigsten Aspekte, die zu einer Reduktion der peri- und postoperativen Mortalität führen.

## Einleitung

Personen im geriatrischen Lebensabschnitt (gr. geron: Alter, Greis) sind die am schnellsten wachsende Untergruppe moderner Gesellschaften, insbesondere in den Industrienationen [1]. Die Zahl der über 65-Jährigen wird zwischen 2000 und 2050 voraussichtlich um 135 % steigen. Die Gruppe der über 85-Jährigen, die am häufigsten Gesundheits- und Langzeitpflegedienstleistungen benötigt, wird in diesem Zeitraum schätzungsweise um 350 % zunehmen. Die Zahl der Hundertjährigen wird im Jahr 2100 weltweit über 25 Mio. betragen [2].

Die Rekonstruktion von **komplexen Weichteildefekten**

komplexen Weichteildefekten

der unteren Extremitäten in der geriatrischen Patientenpopulation ist eine besonders anspruchsvolle orthoplastische Aufgabe. Dies liegt v. a. an der zunehmenden Zahl von Komorbiditäten im Alter, die nicht nur den chirurgischen Eingriff selbst erschweren, sondern eine umfassende peri- und postoperative Behandlung erfordern. Das **orthoplastische Behandlungskonzept**

orthoplastische Behandlungskonzept

impliziert eine gemeinsame, partnerschaftliche Behandlung zwischen Orthopäden/Traumatologen und plastisch-rekonstruktiven Chirurgen sowie ggf. Geriatern und Gefäßchirurgen mit dem Ziel einer möglichst raschen und koordinierten Behandlung für das optimale Ergebnis. Primär soll über den sinnvollen **Extremitätenerhalt**

Extremitätenerhalt

entschieden werden. Hierbei muss berücksichtigt werden, dass eine **Amputation**

Amputation

der unteren Extremität in dieser speziellen Patientenpopulation mit einer erhöhten Mortalität einhergeht [3]. Sollte nach profunder Evaluation der Extremitätenerhalt geplant werden, müssen komplexe Defekte infolge von Tumor, Trauma, Infektion oder Minderdurchblutung anatomisch und funktionell wiederhergestellt werden.

### Fallbeispiel 1

**Figure 1:**
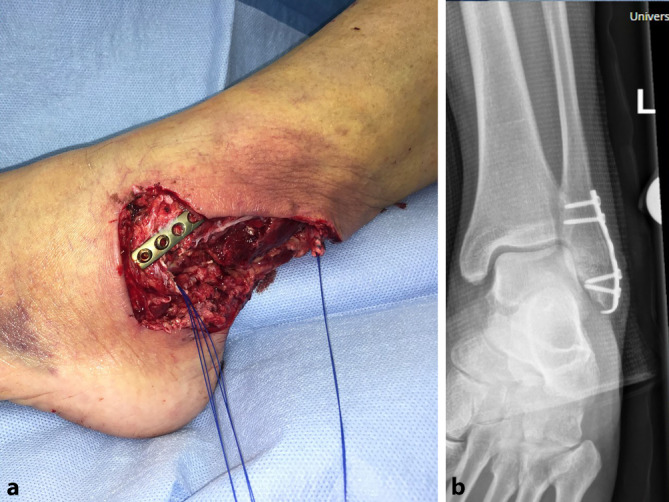

**Figure 2:**
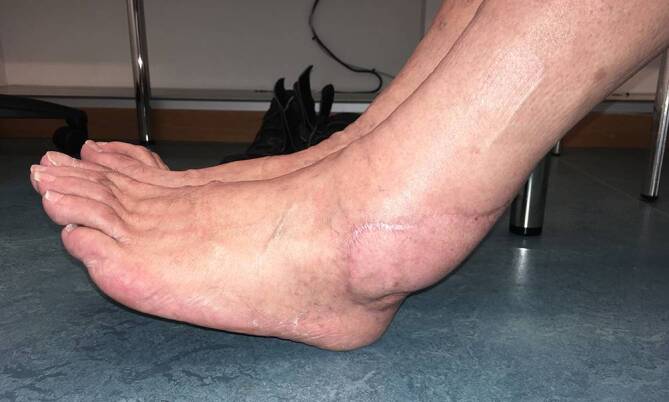

Ein 73-jähriger Patient hatte sich bei Gartenarbeiten mit der Fräse beide Achillessehnen schwer verletzt, linksseitig zusätzlich beide Peronealsehnen durchtrennt und eine offene Fraktur des Caput fibulae zugezogen. Nach unfallchirurgischer Erstversorgung entwickelte sich ein Hämatom mit fulminanter Akutinfektion und Nachweis von *Staphylococcus aureus*. Die angiologische Untersuchung ergab eine mäßige Atherosklerose sowie Abbrüche sowohl der A. tibialis anterior als auch der A. tibialis posterior auf Höhe des oberen Sprunggelenks bei Eingefässversorgung des Fußes über die A. fibularis. Dem Patienten gelang ein kompletter Rauchstopp, und der Ernährungsstatus wurde verbessert. Nach korrektem Débridement zeigten sich ein Weichteildefekt von 6,5 × 17 cm und ein kombinierter Peronealsehnendefekt von 3,8 cm (Abb. 1). In gleicher Sitzung wurde der Defekt mithilfe eines extendierten, sensibilisierten und mit vaskularisierter Trizepssehne augmentierten freien Oberarmlappens rekonstruiert. Durch paralleles Arbeiten betrug die gesamte Zeit der orthoplastischen Operation weniger als 5 h. Die ersten beiden postoperativen Nächte verbrachte der Patient auf der Intensivstation, und ein beginnendes Delir konnte medikamentös rasch behandelt werden. Ab dem 5. postoperativen Tag konnte der Patient unter intensiver physiotherapeutischer Anleitung 2‑mal täglich mit Teilbelastung an Gehstöcken mobilisiert und rasch zum weiteren Kraft- und Ernährungsaufbau in die Rehabilitation verlegt werden. Nach 2 Monaten war es dem geriatrischen Patienten möglich, ohne Gehhilfen voll zu belasten. Alle Wunden waren vollständig verheilt (Abb. 2 und 3), bei uneingeschränkter Funktion (Zusatzmaterial online: Video 1).

**Figure 3:**
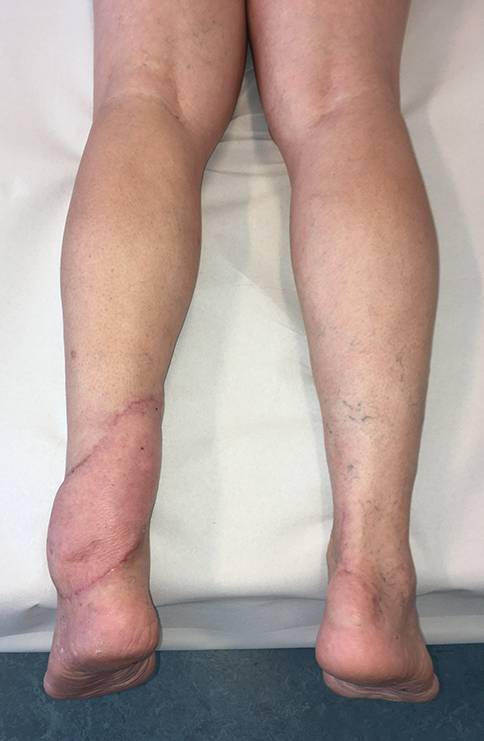

### Fallbeispiel 2

**Figure 4:**
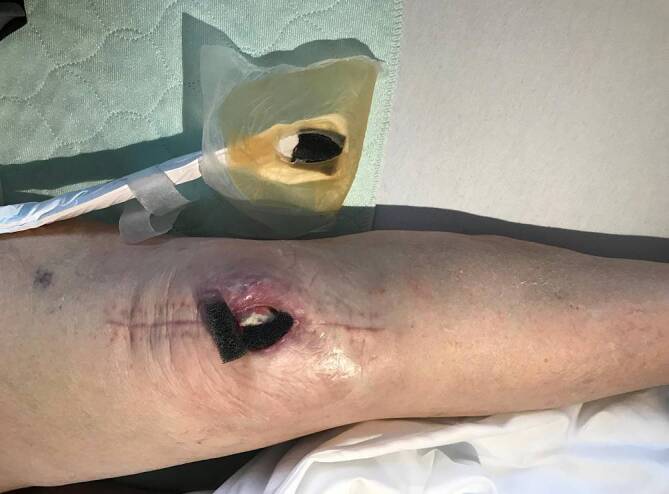

**Figure 5:**
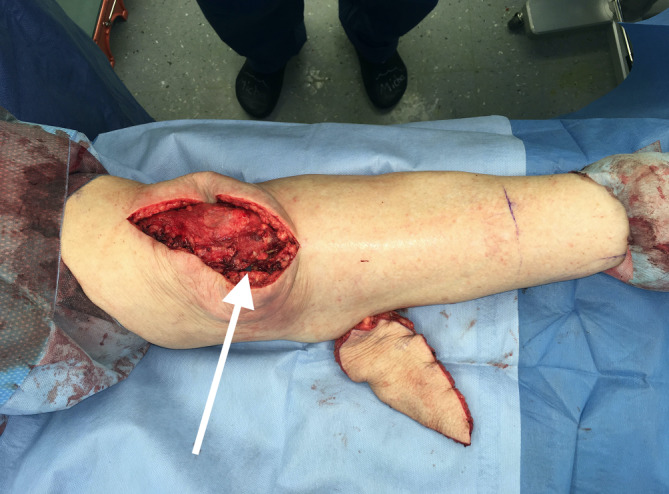

**Figure 6:**
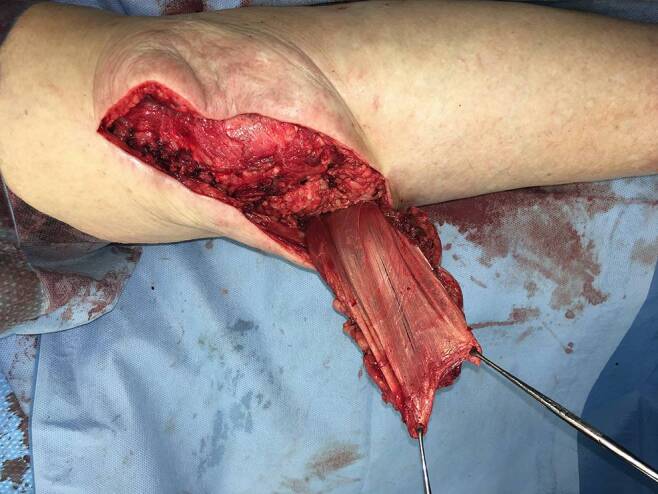

**Figure 7:**
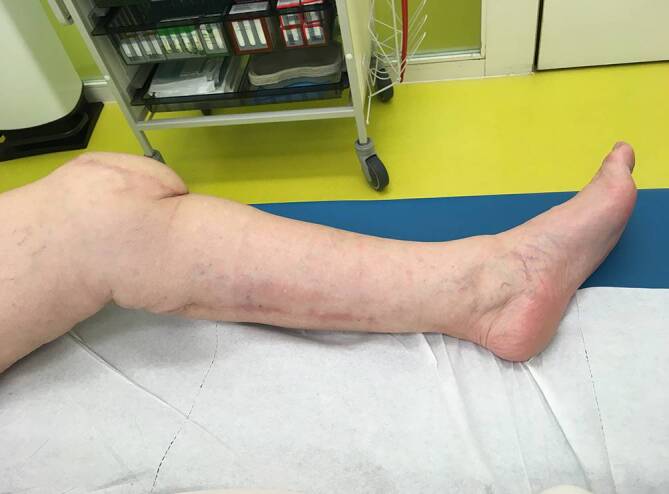

Eine 88-jährige Patientin wies 3 Wochen nach primärem Kniegelenkersatz eine Wundheilungsstörung im Bereich des Zugangs auf. Nach einer Unterdruckbehandlung („negative pressure wound therapy“, NPWT) präsentierte sich insgesamt 5 Wochen nach der Knieprothesenimplantation eine große Fistel mit teilweise nekrotischem Streckapparat (Abb. 4). Wie präoperativ aufgrund der schlechten Weichteilverhältnisse bei Platzhalterimplantation (erster Schritt bei zweizeitigem Wechsel) antizipiert, zeigte die mediale Gelenkkapsel distal einen Kapseldefekt mit exponierter Kunststoffkomponente nach Reimplantation einer neuen Kniegelenkprothese. Dem Prinzip folgend, Gleiches mit Gleichem zu ersetzen, wurde bei der Reimplantation (zweiter Schritt) ein gestielter, myokutaner, medialer Gastrocnemiuslappen gehoben (Abb. 5). So konnten die Gelenkkapsel mit der tendinösen Rückfläche des Gastrocnemiuslappens (Abb. 6) und die Haut mithilfe einer perforatorbasierten Hautinsel rekonstruiert werden. Drei Monate postoperativ war eine restitutio ad integrum mit wenig auftragender Hautinsel des Lappens und direkt verschlossener Entnahmestelle erreicht (Abb. 7). Die Kniefunktion betrug Flexion/Extension 130-0-10° (Zusatzmaterial online: Video 2).

## Präoperatives Management

### Präoperative Patientenbeurteilung

In der präoperativen Beurteilung ist es beim alten Patienten umso wichtiger, zwischen dem technisch Machbaren sowie dem medizinisch und ethisch Sinnvollen abzuwägen. Folgende Aspekte müssen in der präoperativen Abklärung beachtet werden:

- Komorbiditäten, v. a. Erkrankungen von Herz und Kreislauf, Lungen, Nieren und Leber,
- Gefäßstatus (*Cave*: Atherosklerose),
- Einschränkungen der Wundheilung (Diabetes, Autoimmunerkrankungen),
- Ernährungsstatus (Proteinmangel, v. a. Albumin hinsichtlich Wundheilung, Kalorienbedarf),
- Prehabilitation.

Zur **ganzheitlichen Behandlung**

ganzheitlichen Behandlung

des Patienten sollten auch Spezialisten anderer Fachgebiete einbezogen werden. Dies ermöglicht eine raschere Versorgung und eine frühere Rehabilitation und verbessert beim geriatrischen Patienten nicht nur die funktionellen klinischen Ergebnisse, sondern senkt auch die längerfristige Mortalität erheblich [4, 5]. In Tab. 1 findet sich eine stichwortartige Zusammenfassung der relevanten Aspekte, die im Behandlungsteam vor Beginn der Behandlung diskutiert und geplant werden sollen.

**Table 1:** 

Fragestellung	Wichtige Gesichtspunkte
Fragen bei der Indikationsstellung im Alter	Erwartungen/Wünsche des Patienten, physische Belastung, Lebensqualität, Komorbiditäten, alternative Therapie, Rückzugsoptionen, Interdisziplinarität, Ethikrat
Operationsvorbereitung	Detaillierte, interdisziplinäre Planung, spezifische Gefäßdiagnostik u. a. Duplexsonographie, Angiographie (CT-/MR-Angiographie, konventionell, interventionell), Phlebographie, Notwendigkeit eines AV-Loop/Bypasses prüfen, Anwendung von zuverlässigen Standardtechniken („workhorse flaps“), Patientenverfügung/Vorsorgevollmacht
Komorbiditäten und perioperatives Management	Relevanz kardiologischer, nephrologischer, pneumologischer, hämostaseologischer Komorbiditäten, Kooperation mit der Geriatrie etablieren, Anwendung von „frailty assessments“, Möglichkeit der Prehabilitation, Minimierung der anästhesiologischen Belastung, der Anzahl notwendiger Operationen sowie der Operationszeit, Vermeidung eines Lehreingriffs
Besonderheiten postoperative Phase	Leitlinien/Konsensus bezüglich Perfusionsmonitoring, Schmerztherapie, Gerinnungsmanagement, intensivierter Dekubitusprophylaxe, rascher Mobilisierung, Fortbildung der Ärzte/des Pflegepersonal bezüglich Versorgung alter Patienten, Delirprophylaxe/-therapie

*AV* arteriovenös

Bei **Stürzen**

Stürzen

(häufigste Unfallursache beim geriatrischen Patienten) sollte eine ausführliche Analyse zum Ausschluss neurologischer oder kardiologischer Ursachen durchgeführt werden, z. B. durch EEG, EKG, Belastungs-EKG und transösophageale Echokardiographie (TEE).

Präoperativ sollten routinemäßig **laborchemische Untersuchungen**

laborchemische Untersuchungen

des Elektrolyt- und Glucosehaushalts, Blutbilds (Hämoglobin, Leukozyten- und Thrombozytenzahl), sowie der Nierenfunktion erfolgen, ebenso der Ausschluss einer **Mangelernährung**

Mangelernährung

(Gesamteiweiß‑, Albumin- und Vitaminspiegel).

Ältere Patienten sind häufig mit **Vitamin-K-Antagonisten**

Vitamin-K-Antagonisten

oral antikoaguliert, daher muss die **Blutgerinnung**

Blutgerinnung

präoperativ kontrolliert und angepasst werden. Aufgrund vielfacher Wechselwirkungen mit anderen Antikoagulanzien und weiteren Medikamenten sollte niederschwellig ein Hämatologe involviert werden [4, 6].

Der erste Schritt zum Ausschluss einer relevanten peripheren arteriellen Verschlusskrankheit (pAVK) bleibt die Palpation der **distalen Fußpulse**

distalen Fußpulse

(Knöchel-Arm-Index: Quotient aus Blutdruck am Unterschenkel und Blutdruck am Oberarm zur Abschätzung einer Durchblutungsstörung im Seitenvergleich), da bei gut palpablen distalen Pulsen eine relevante PAVK sehr unwahrscheinlich ist [7].

Insbesondere vor mikrochirurgischen Eingriffen ist sicherheitshalber eine genauere **Gefäßdiagnostik**

Gefäßdiagnostik,

zumindest mithilfe der **Duplexsonographie**

Duplexsonographie,

zu empfehlen [8]. Sollten bereits **Wundheilungsstörungen**

Wundheilungsstörungen

oder **Nekrosen**

Nekrosen

vorliegen, ist von einer „critical limb ischemia“ (Fontaine-Stadium IV [9]) auszugehen sowie eine angiologische Untersuchung und bildgebende Untersuchung (CT- oder MRI-Angiographie) unabdingbar. Stellen sich darin relevante Gefäßeinschränkungen dar, sollten nach Bedarf die Kollegen der Angiologie, interventionellen Radiologie oder Gefäßchirurgie hinzugezogen werden, um interdisziplinär z. B. eine präoperative Stent-Einlage/Dilatation oder eine Bypass‑/Loop-Anlage zu besprechen.

### Klassifizierung – Alter vs. Gebrechlichkeit

In der Literatur ist die Definition von „alt“ sehr heterogen und hat sich im Laufe der Zeit stark verändert – früher galten Patienten bereits ab 50 Jahren als „alt“. Heute existieren verschiedene Klassifizierungen, wie z. B. jung-alt (> 65 Jahre) vs. alt-alt oder sehr alt (> 80 Jahre); dazu noch die sog. **Supersenioren**

Supersenioren

(90 bis 100 Jahre) und die **„supercentenarians“**

„supercentenarians“

(> 110 Jahre; [10, 11, 12]). Entscheidend erscheint das **biologische Alter**

biologische Alter

und nicht das chronologische. Beim Fokus auf das biologische Alter werden **biophysiologische Messungen**

biophysiologische Messungen

durchgeführt, um das mit dem Alter steigende Risiko für medizinische Zwischenfälle zu berechnen („adverse events“ oder „adverse outcomes“, [13]). Ausschlaggebend sind verschiedene biologische Faktoren, wie beispielsweise der vaskuläre Ab- und Umbau sowie verschiedene systemische Biomarker [14].

Hinzukommt der Begriff der Gebrechlichkeit, englisch **„frailty“**

„frailty“,

definiert „als Akkumulation von Defiziten, welche den Patienten in medizinischen Notsituationen vulnerabler machen“ [15]. Dieser Ansatz bietet im Gegensatz zum rein chronologischen Alter den großen Vorteil, dass unmittelbare Rückschlusse auf die Behandlung und die Prognose des Patienten möglich sind [13, 16, 17]. Geriatrische Patienten sind demnach untereinander eine heterogene Gruppe, da hohes Alter nicht mit denselben Komorbiditäten und einheitlicher Lebenserwartung einhergeht. Bei geriatrischen Patienten ist es wichtig, dass der Behandler (Chirurg) den Patienten in seiner Ganzheitlichkeit kennt, um die entsprechende(n) interdisziplinäre(n) Zusammenarbeit(en) zu veranlassen.

#### Merke

Gebrechlichkeit und biologisches Alter erlauben, im Gegensatz zum chronologischen Alter, einen Rückschluss auf die Prognose des Patienten.

## Allgemeine Kontraindikationen gegen rekonstruktive Eingriffe

Kontraindikationen gegen Eingriffe zur Wiederherstellung der unteren Extremität beinhalten:

- allgemeine Inoperabilität (schlechter Allgemeinzustand, Bettlägerigkeit, sehr kurze Lebenserwartung, hohes Risiko für schwere Morbidität/Mortalität),
- knöcherne Stabilisierung nicht möglich,
- keine adäquaten Spendergefäße verfügbar/rekonstruierbar (Revaskularisation nicht möglich),
- mangelnde Compliance (z. B. Drogenmissbrauch, psychiatrische/demenzielle Erkrankungen).
- Bei malignen Extremitätentumoren muss in einer potenziell kurativen Situation, unabhängig vom Alter des Patienten, bei gegebener Operabilität onkologisch radikal therapiert werden. Vermeidungsstrategien und vermeintliche kleinere chirurgische Lösungen führen häufiger zu Komplikationen, z. B. Wundheilungsstörungen, verzögerter Rehabilitation und Lokalrezidiven. Eine isolierte Radiatio kann erwogen werden, wenn bereits Fernmetastasen vorliegen und keine kurative Intention mehr besteht.

## Behandlungsziele

Primäres Ziel ist die Wiederherstellung der **Beinfunktion**

Beinfunktion

– schmerzfreies Stehen und Gehen – und der **Mobilität**

Mobilität,

um die Eigenständigkeit des alten Patienten zu wahren. Zur Prävention von Stürzen sind **Propriozeption**

Propriozeption

und **Sensibilität**

Sensibilität

notwendig; eine Sturzneigung kann auch durch ein Nervenkompressionssyndrom bedingt sein (N. tibialis und N. peroneus communis; [18]). Lange Hospitalisationen infolge von Stürzen haben oft weitreichende Folgen (z. B. Pneumonie, Thrombose, Lungenembolie), die letal enden [19, 20] können.

### Merke

Ziele der Behandlung sind die schmerzfreie Geh- und Stehfunktion sowie der Erhalt von Mobilität und Autonomie.

## Entscheidungsfindung und Behandlungsalgorithmus

**Figure 8:**
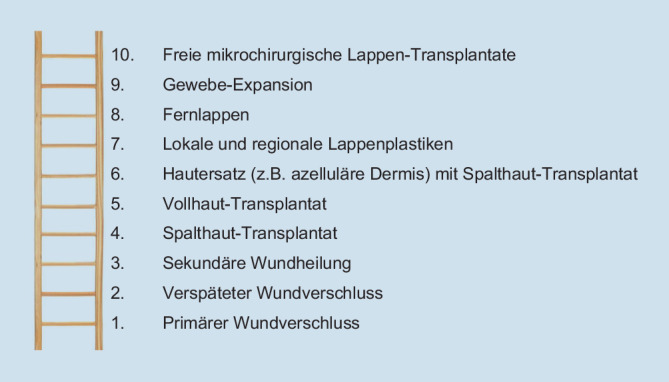

Die individuelle Verfahrenswahl orientiert sich klassischerweise am Konzept der **plastisch-rekonstruktiven Stufenleiter**

plastisch-rekonstruktiven Stufenleiter

(Abb. 8). Diese führt vom Direktverschluss über die Hauttransplantation zur lokalen und schließlich zur freien Lappenplastik („so aufwendig wie nötig, so einfach wie möglich“). Um das Ziel einer passgenauen Rekonstruktion hinsichtlich Form, Funktion und Ästhetik durch Gewebe mit ähnlichen Eigenschaften („Gleiches durch Gleiches ersetzen“) zu erreichen, müssen gemäß dem moderneren Konzept des **plastisch-rekonstruktiven Aufzugs**

plastisch-rekonstruktiven Aufzugs

bei Bedarf Stufenleitersprossen übersprungen werden. Als Beispiel ist die Spalthauttransplantation auf Muskel zu erwähnen, die häufig zu störenden Einziehungen, instabilen Narben und nicht selten neuropathischen Schmerzen führt, zu erwähnen. Dies kann beispielsweise am Fuß, der mechanisch stark belastet wird, chronische Probleme bedingen. Eine **mikrochirurgische Lappenplastik**

mikrochirurgische Lappenplastik

erlaubt hingegen eine passgenaue, sensibilisierte Rekonstruktion eines dreidimensionalen Defekts, einschließlich Plombierung von Totraum, sowie einen spannungsfreien und belastungsfähigen Wundverschluss. Die robustere **Lappenperfusion**

Lappenperfusion

ermöglicht zusätzlich eine effektivere antibiotische Infektionsbehandlung. Die wichtigsten Verfahren zur lokalen und zur freien Lappenplastik sind in Tab. 2 wiedergegeben.

**Table 2:** 

	Gefäßversorgung	Indikation	Vorteile	Nachteile	Bemerkungen
*Lokale Lappenplastik*					
Medialer/lateraler M.-gastrocnemius-Lappen (Muskellappen)	A. suralis medialis/lateralis	Weichteildefekte des distalen Oberschenkels und Knies sowie des proximalen Drittels des Unterschenkels	Leichte, schnelle Präparation	Häufig Notwendigkeit einer Spalthautdeckung	Bei traumatischen Verletzungen präoperative angiologische Untersuchung zum Ausschluss einer relevanten arteriellen/venösen Durchblutungsstörung sinnvoll
			Zuverlässige Blutversorgung		
			Kein Umlagern notwendig, kann gut in Rückenlage des Patienten gehoben werden		
			Kann um eine perforatorbasierte Hautinsel erweitert werden		
			Minimale funktionelle Einschränkung		
			Tendinöse Rückseite des Muskellappens kann zu Kapsel- und Streckapparatrekonstruktion verwendet werden		
Arteria-suralis-medialis-Perforator-(engl. medial sural artery Perforator, MSAP)-M.-gastrocnemius-Lappen (Myokutaner Lappen)	Perforator der A. suralis medialis	Weichteildefekte des distalen Oberschenkels und Knies sowie des proximalen Drittels des Unterschenkels	Zuverlässige Blutversorgung	Bei traumatischen Verletzungen können die Hautperforatoren geschädigt sein	Erlaubt eine direkte Hautnaht der Hautinsel im Defektbereich (vorteilhaft speziell bei implantatassoziierten Infektionen, bei denen rasch trockene Wundverhältnisse erwünscht sind
			Kein Umlagern notwendig, kann gut in Rückenlage des Patienten gehoben werden		
			Häufig Direktverschluss der Entnahmestelle möglich		
			Minimale funktionelle Einschränkung		
			Tendinöse Rückseite des Muskellappens kann zu Kapsel- und Streckapparatrekonstruktion verwendet werden		
Arteria- suralis-lateralis-Perforator-(engl. lateral sural artery perforator, LSAP)-M.-gastrocnemius-Lappen (Myokutaner Lappen)	Perforator der A. suralis lateralis	Weichteildefekte des lateralen Knies und des proximalen Unterschenkel-Drittels	Kein Umlagern notwendig, kann gut in Rückenlage des Patienten gehoben werden	Gelegentlich kein Perforatorgefäß vorhanden	Erlaubt eine direkte Hautnaht der Hautinsel im Defektbereich (vorteilhaft speziell bei implantatassoziierten Infektionen, bei denen rasch trockene Wundverhältnisse erwünscht sind)
			Häufig Direktverschluss der Entnahmestelle möglich	Durch den Lappenzug kann der N. peronaeus communis eingeengt werden	
			Minimale funktionelle Einschränkung	Durch kürzeren Muskelbauch und zusätzlichen Weg um die Fibula reicht der Lappen deutlich weniger weit als der MSAP-Gastrocnemius-Lappen	
			Tendinöse Rückseite des Muskellappens kann zu Kapsel- und Streckapparatrekonstruktion verwendet werden	Bei traumatischen Verletzungen können die Hautperforatoren geschädigt sein	
Geteilter („split“)-M.-soleus-Lappen	Äste aus der A. tibialis posterior	Kleinere Weichteildefekte im mittleren Unterschenkelbereich	Kein Umlagern notwendig, kann gut in Rückenlage des Patienten gehoben werden	Kleiner Rotationsradius	Bei traumatischen Verletzungen präoperative angiologische Untersuchung zum Ausschluss einer relevanten arteriellen/venösen Durchblutungsstörung sinnvoll
			Direktverschluss der Entnahmestelle möglich	Perfusionsstörungen der Lappenspitze	
			Minimale funktionelle Einschränkung	Benötigt Spalthautdeckung	
M.-peroneus-brevis-Lappen (Muskellappen)	Äste aus A. fibularis und A. tibialis anterior	Kleine Weichteildefekte der distalen Fibula	Minimale funktionelle Einschränkung	Kleiner Lappen mit geringer Reichweite	–
			Direktverschluss der Entnahmestelle	Benötigt Spalthautdeckung	
				Häufig Perfusionsstörung der Lappenspitze (venöse Stauung)	
Suralis-Lappen (Fasziokutaner Lappen)	Neurovaskulärer Lappen des N. suralis und seiner Begleitgefässe (A. suralis und Venen)	Weichteildefekte des Unterschenkels, des Sprunggelenks, der Ferse und des Fußes	Großer Rotationsradius	Perfusion bei pAVK und nach Trauma oft unzuverlässig	Komplikationsraten bei älteren Patienten sehr hoch, v. a. durch Lappenspitzennekrose
			Rasche Präparation	Häufig Durchblutungsstörung der Lappenspitze (venöse Stauung)	Anspruchsvolle postoperative Hochlagerung der Ferse
				N. suralis muss „geopfert“ werden	
				Entnahmestelle kann häufig nicht direkt verschlossen werden	
*Mikrochirurgische Lappenplastik*					
M.-gracilis-Lappen (Muskellappen)	A. circumflexa femoris medialis	Kleine bis mittelgroße Weichteildefekte am Unterschenkel	Zuverlässiger anatomischer Zustand der Gefäße	Kann um Hautinsel erweitert werden	Sensibler Anschluss über den N. obturatorius möglich
			Geringer Hebedefekt	Benötigt Spalthautdeckung	Kann auch als sehr kleiner Lappen („nugget“) gehoben werden
			Unauffällige Narbe		
Anterolateraler Oberschenkellappen (ALT) (Fasziokutaner Lappen)	R. descendens der A. circumflexa femoris lateralis	Mittelgroße bis große Weichteildefekte am Unterschenkel	Gut form- und ausdünnbar	Lange Narbe an der Entnahmestelle	An der Klinik der Autoren der Standardlappen zur Unterschenkelrekonstruktion
			Großer Lappen	Kann bei adipösen Patienten dick und deshalb schlecht faltbar sein	Sensibler Anschluss über N. cutaneus femoris lateralis möglich
			Langer, kaliberstarker Stiel		
			Geringer Hebedefekt		
			Minimale funktionelle Einschränkung		
			Kann als chimärer Lappen gehoben werden (mit Fascia lata bei Sehnenverletzungen/mit M. vastus lateralis bei ausgedehnten Defekten)		
			Kann als Durchflusslappen bei Gefäßdefekten gehoben werden		
Fasziokutaner lateraler Oberarmlappen (Fasziokutaner Lappen)	A. collateralis radialis aus A. profunda brachii	Kleine bis mittelgroße Weichteildefekte am Unterschenkel, insbesondere für Defekte um Achillessehne und Malleolus geeignet	Sehr dünner und somit gut faltbarer Lappen	Kurzer Stiel	Sensibler Anschluss über N. cutaneus brachiilateralis möglich
			Kann nach distal extendiert gehoben werden	Hyposensibilität im Bereich der Entnahmestelle/des Unterarms	
			Kann mit Trizepssehne gehoben werden		
Leistenlappen (Fasziokutaner Lappen)	A. circumflexa ilium superficialis	Kleine bis mittelgroße Weichteildefekte am Unterschenkel, insbesondere für Defekte um die Achillessehne und den Malleolus geeignet	Sehr dünner und somit gut faltbarer Lappen	Kurzer Stiel	Historisch die erste erfolgreiche freie Lappenplastik [22]
			Entnahmestelle kann direkt verschlossen werden		
Perforator-Leisten-Lappen (SCIP) (Fasziokutaner Lappen)	A. circumflexa ilium superficialis Perforator	Kleine bis mittelgroße Weichteildefekte am Unterschenkel, insbesondere für Defekte um die Achillessehne und den Malleolus geeignet	Sehr dünner und somit gut faltbarer Lappen	Kurzer Stiel	Weiterentwicklung des Leistenlappens
			Entnahmestelle kann direkt verschlossen werden		
M.-latissimus-dorsi-Lappen (Muskellappen)	A. thoracodorsalis	Große Weichteildefekte der unteren Extremität	Größter freier Lappen	Benötigt meist Spalthautdeckung	–
			Gute Faltbarkeit, kann auch für zirkumferenzielle Defekte benutzt werden		
			Kann mit Hautinsel gehoben werden		
			Minimale funktionelle Einschränkung		
Medialer Femurkondyluslappen (Knochenlappen)	A. genus descendens und A. superior medialis genus	Kleine Knochendefekte der unteren Extremität	Zuverlässige anatomische Verhältnisse, gute Knochenqualität	Limitierte Größe	Kann auch als reiner Periostlappen für atrophe Pseudarthrosen gehoben werden
			Geringe Hebemorbidität		

*pAVK* periphere arterielle Verschlusskrankheit

### Merke

Die Entscheidungsfindung folgt dem Prinzip der „rekonstruktiven Stufenleiter“ oder dem moderneren Ansatz des „rekonstruktiven Aufzugs“.

## Chirurgische Planung und Durchführung

Geriatrische Patienten tolerieren Stress, hohe intraoperative Belastung und postoperative Komplikationen schlechter, da ihre Organreserven geringer sind. Deswegen muss die Zahl der Narkosen auf ein Minimum reduziert und die am wenigsten belastende Narkosetechnik (wenn möglich regionale Anästhesie in weitgehend sitzender Position) gewählt werden [23]. Das chirurgische Vorgehen sollte, wenn möglich, einzeitig sein. Dies ist nur mithilfe fundierter, interdisziplinärer, präoperativer Planung möglich. So können die verschiedenen intraoperativen Befunde antizipiert sowie Lösungswege präoperativ diskutiert und festgelegt werden. Die **Operationszeit**

Operationszeit

muss auf ein Minimum reduziert werden. Dies kann durch das Arbeiten mehrerer Teams mit erfahrenen Chirurgen, die z. B. gleichzeitig an Hebe- und Empfängerstelle arbeiten, erreicht werden. Ferner sollten diese Eingriffe nicht als langwierige Lehreingriffe dienen. Offene Wunden führen die Patienten in einen **katabolen Metabolismus**

katabolen Metabolismus,

weshalb die Rekonstruktion so rasch als möglich erfolgen sollte.

### Merke

Der geriatrische Patient erfordert einen adaptierten Behandlungsablauf – je rascher und koordinierter die Behandlung, desto besser.

### Lokoregionale Lappen

Genaueres Wissen über die Gefäßanatomie bis auf die Ebene der Perforatoren, auch bedingt durch neue hochauflösende Bildgebung (CT- und MR-Angiographie, Duplexsonographie) und neue chirurgische Techniken, hat die Optionen der lokalen und regionalen Lappen für die Rekonstruktion der unteren Extremität wesentlich erweitert. Sie kommen grundsätzlich für kleinere (< 50 cm^2^) und einfachere Wunden in Betracht, wenn ausreichend Gewebe außerhalb der Verletzungszone mobilisiert werden kann. **Perforatorbasierte Lappen**

Perforatorbasierte Lappen

können abhängig von lokal festgestellten Gefäßen individuell angepasst werden („free style perforator flaps“). Ein wesentlicher Vorteil ist die meist deutlich kürzere Operationszeit.

### Mikrochirurgie

Die Erfolgsrate freier Lappen beträgt heute über 90 %, sodass diese auch bei alten Patienten indiziert sein können. Spezielle Risiken ergeben sich bei ausgeprägten Gefäßerkrankungen, jedoch ist die Gefahr von Wundheilungsstörungen und Nekrosen in diesen Fällen bei lokalen Lappenplastiken ebenfalls gegeben. Wenn trotz gefäßchirurgisch/angiologischer Intervention keine verbesserte Perfusion erzielt werden kann, können Veneninterponate, Bypässe oder arteriovenöse (AV‑)Loops zum Anschluss des freien Lappens als Alternative zur Amputation diskutiert werden.

Das Argument gegen eine mikrochirurgische Rekonstruktion bei hochbetagten Patienten war früher v. a. die hohe Belastung infolge der langen Operationsdauer in Vollnarkose. Heute sind solche Operationen aufgrund des anästhesiologischen und chirurgischen Fortschritts zuverlässiger planbar und sicher und weisen eine deutlich reduzierte perioperative Morbidität und Mortalität auf [24]. Die Mikrochirurgie ist heute nicht mehr Ultima Ratio (s. auch rekonstruktiver Aufzug [25]), wenn alles andere versagt hat, sondern wird oft als **komplikationsarmes Routineverfahren**

komplikationsarmes Routineverfahren

für einzeitige Rekonstruktionen bevorzugt, z. B. in der orthoplastischen Chirurgie [26, 27, 28, 29].

Das Vorgehen in 2 OP-Teams (gleichzeitige Vorbereitung der Empfängerstelle, Lappenhebung und Verschluss der Spenderstelle) sowie die Verwendung schneller und zuverlässiger Techniken mit langen und konstanten Gefäßen aus der unteren Extremität (z. B. Grazilislappen oder anterolateraler Oberschenkellappen [ALT]) reduzieren Operationszeit und die operative Belastung des Patienten. Bei stark atherosklerotischen Gefäßen sollten 8/0- oder 7/0-Nähte mit stärkeren Nadeln und möglichst keine Klemmen am Gefäß verwendet werden; gute Alternativen sind **Tourniquets**

Tourniquets

oder **intraluminale Verschlussvorrichtungen**

intraluminale Verschlussvorrichtungen.

Freie, mikrochirurgische Lappenplastiken sind besonders geeignet für:

- ausgedehnte Weichteildefekte der unteren Extremität, komplexe osteo- und tenofasziokutane Defekte sowie Defekte des Kniestreckapparats oder freiliegende Knie- und Sprunggelenkprothesen;
- Unterschenkelverletzungen nach hochenergetischem Trauma,
- offene Frakturen: Tibiafrakturen des Grades III mit erheblichem Gewebeverlust und damit verbundenem hohen Risiko für Infektionen, Pseudarthrosen, verlängertem Krankenhausaufenthalt und Amputation – mithilfe lokaler Lappen kann oft keine suffiziente Deckung erreicht werden;
- chronische Osteomyelitis (z. B. durch Trauma oder Diabetes, mit multiplen resistenten Keimen, Knochendefekten, ausgedehnter Narbenbildung und Fibrose aus früheren Behandlungsversuchen, die häufig Kontraindikationen für die Anwendung von lokalen Lappen darstellen);
- frakturassoziierte Infektion und periprothetische Gelenkinfektion;
- Fuß- und Sprunggelenkdefekte (mit frei liegenden Knochen und Sehnen oder Verlust der belasteten Fußsohlenfläche, die lokale Lappen ausschließen, eine längere Ruhigstellung und einen protrahierten Krankenhausaufenthalt verursachen können, z. B. aufgrund von Mehrfachoperationen);
- onkologische Resektionen (z. B. bei Weichteilsarkom), bei denen komplexe Weichteildefekte entstehen [30].

## Postoperative Versorgung und erwartetes Ergebnis

Engmaschige Kontrollen auf der Intensivstation sind häufig notwendig, um bei Komplikationen sofort eingreifen zu können. Bei älteren Patienten wurde ein Trend zu nicht direkt operationsbedingten Komplikationen beobachtet, v. a. an Herz (Infarkt), Lungen (Pneumonie, Embolie) und Nieren. Eine **intensivmedizinische Überwachung**

intensivmedizinische Überwachung

über 12–48 h erlaubt:

- Lappenüberwachung zur Früherkennung von Perfusionsproblemen,
- restriktives Flüssigkeits- und Blutdruckmanagement (Vasoaktiva),
- Aufrechterhaltung der Körpertemperatur,
- optimale Ernährung,
- spezialisierte Pflege und Physiotherapie, frühzeitige Mobilisierung, einschließlich Atemgymnastik und Lappentraining,
- Schmerzkontrolle,
- Delirprophylaxe.

## Komplikationsmanagement

Komplikationsraten, Mortalität und Morbidität können durch die oben genannten Konzepte reduziert werden [8]. Früherkennung ist wichtig, um eine schnelle Verschlechterung des Zustands eines älteren Patienten mit **geringerer Organsystemreserve**

geringerer Organsystemreserve

und geringerer Komplikationstoleranz zu vermeiden. Allgemeine Komplikationen der Organfunktionen und des mentalen Status sollten multidisziplinär angegangen werden. Lokale Komplikationen an der Spender- und Empfängerstelle sollten rasch chirurgisch behandelt werden.

## Fazit für die Praxis

- Der stetig steigende Anteil älterer Menschen (jenseits des 7. Lebensjahrzehnts) an der Bevölkerung in vielen modernen Gesellschaften geht mit einem erhöhten Bedarf an komplexen Rekonstruktionen der unteren Extremitäten einher.
- Die Rekonstruktion der unteren Extremität kann bei älteren Patienten sicher, mit hoher Erfolgsrate und überschaubaren Komplikationen durchgeführt werden. Mikrochirurgische Verfahren sind aufgrund des fortgeschrittenen Alters nicht per se kontraindiziert (biologisches vor chronologischem Alter); vielmehr dienen sie in geeigneten Fällen dazu, die Gehfähigkeit, Mobilität, Autonomie und Lebensqualität der älteren Menschen zu erhalten.
- Zu den Voraussetzungen gehören: umfassende präoperative, interdisziplinäre Beurteilung des Patienten sowie Optimierung der Risikofaktoren wie z. B. Diabetes, Mangelernährung, verminderte Extremitätenperfusion, gestörte Nieren- und Herzfunktion, sorgfältige präoperative interdisziplinäre peri- und operative Planung, intraoperative Anpassung der Anästhesie sowie adaptierte und effiziente Operationstechnik, spezialisierte postoperative Überwachung und frühzeitige Mobilisierung entsprechend den spezifischen Bedürfnissen des Patienten im fortgeschrittenen Alter.

### Supplementary Information






## CME-Fragebogen

Welcher Gesichtspunkt spielt bei der Indikationsstellung und Vorbereitung komplexer rekonstruktiver Eingriffe bei Patienten im hohen Lebensalter eine entscheidende Rolle?

Die individuellen Erwartungen des Patienten spielen eine untergeordnete Rolle.

Die Durchführung sollte in mehrere operative Eingriffe und Narkosen aufgeteilt werden.

Interdisziplinäre Entscheidungsfindungen sollten vermieden werden, da dies den Prozess verkompliziert.

Spezifische Bildgebungstechniken, z. B. Duplex, Angiographie zum Ausschluss von Gefässpathologien sind empfehlenswert.

Bei Patienten mit einem chronologischen Alter über 90 Jahren sollten primär lokale Lappenplastiken eingesetzt werden.

Was sollte bei lokalen und mikrochirurgischen Lappenplastiken zur Rekonstruktion der unteren Extremität beachtet werden?

Die mikrochirurgischen Lappenplastiken erlauben es heute Defekte von maximal 10 x 10 cm zu decken.

Perforator-basierte lokale Lappen erfordern aufgrund der diffizilen Präparationstechnik meist längere Operationszeiten als mikrochirurgische Lappenplastiken.

Die liegende ist der sitzenden Lagerung bei älteren Patienten vorzuziehen, da sie weniger kreislaufbelastend ist.

Rekonstruktive mikrochirurgische Eingriffe bei geriatrischen Patienten sind als Lehreingriffe für jüngere Kollegen geeignet, wenn sie in Vollnarkose durchgeführt werden.

Offene Wunden führen ältere Patienten oft in einen katabolen Metabolismus, weshalb die Rekonstruktion raschmöglichst erfolgen sollte.

Für welche Situation eignet sich die lokale Lappenplastik besonders gut?

Komplexe Defekte des Knie-Streckapparates oder freiliegende Knie- und Sprunggelenks-Prothesen

Hochrasanzverletzungen am Unterschenkel, z. B. Autoanprallverletzungen

Verzögerte Knochenheilungen und etablierte Pseudarthrosen, da sie die regionale Perfusion weniger stören

Postonkologische Resektionen, da hier mikrochirurgische Techniken nur als Ultima ratio eingesetzt werden sollten

Wenn sie aufgrund von festgestellten Gefässen vor Ort individuell an eine Defektsituation angepasst werden können („Free style perforator flaps“).

Bei einer 89-jährigen Patientin mit infizierter prätibialer Wundheilungsstörung und schwerer COPD bei fortgesetztem Rauchen dienen welche der folgenden Massnahmen einer Optimierung von OP-Risiken?

Eine Internistische Einstellung mit oralem Vitamin-K-Antagonisten, um Thromboserisiko zu senken.

Eine langfristige Wundkonditionierung, z. B. mit Vakuum-Systemen, um die Keimlast zu senken und die Granulation anzuregen.

Die Gefäßdiagnostik mit angiologischer Untersuchung um bei relevanten Perfusionsblockaden die Revaskularisation durchzuführen.

Die Unterschenkelamputation, um die Operationsbelastung möglichst zu minimieren.

Eine Rauchstop-Beratung ist bei dieser Patientin sinnlos, da Sie schon seit vielen Jahrzehnten raucht.

Was sollte in der Behandlungsstrategie bei der Wiederherstellung der unteren Extremität bei geriatrischen Patienten am ehesten beachtet werden?

Die «rekonstruktive Stufenleiter» spielt heute eine untergeordente Rolle.

Die Bedeutung des individuellen Behandlungsschemas nimmt mit zunehmendem Alter ab.

Die Rekonstruktion der Funktion spielt im geriatrischen Patienten eine geringe Rolle.

Oft wird der «rekonstruktive Aufzug» gewählt, um ein adäquates anatomisches und funktionelles Ziel zu erreichen

Es sollten in erster Linie die chirurgischen Disziplinen miteinbezogen werden, damit das Behandlungsschema schnell ablaufen kann.

Worauf sollte bei der Überprüfung der Durchblutung der unteren Extremität besonders geachtet werden?

Die klinische Untersuchung mit Palpation der Fusspulse ist wenig aussagekräftig.

Eine relevante Durchblutungsstörung wird in der Regel mit invasiven diagnostischen Mitteln dargestellt.

Die Kollegen der Angiologie sollten frühzeitig hinzugezogen werden.

Die Entscheidung bzgl. Diagnostik und ggf. vaskulären Intervention wird durch den behandelnden Chirurgen getroffen.

Es sollte unmittelbar eine MRT-Angiographie durchgeführt werden.

Weshalb stellt die Rekonstruktion der unteren Extremität bei geriatrischen Patienten eine besondere Aufgabe dar?

Die Amputation der unteren Extremität geht mit einer erhöhten Mortalität einher.

In der Regel ist eine mikrochirurgische Rekonstruktion durchzuführen.

Aufgrund der kurzen Operationsdauer eignen sie sich als Lehroperationen.

Die Rekonstruktion stellt einen «ultima ratio» Eingriff dar.

Eine postoperative Mobilisation des Patienten ist häufig nicht mehr möglich.

Worauf sollte bei der Wiederherstellung der unteren Extremitäten bei geriatrischen Patienten geachtet werden?

Die Propriozeption und Sensibilität spielen bei der Planung der Rekonstruktion eine untergeordnete Rolle.

Die Bettlägerigkeit kann vernachlässigt werden, da der metabolische Umsatz gering ist.

Die Wund- und Knochenheilung ist beim geriatrischen Patienten besser, da diese eine höhere Compliance aufweisen.

Rekonstruktive Verfahren mit Bypässen und AV-loops können als Alternativen zur Amputation diskutiert werden.

Komplexe Operationen verbessern wesentlich das postoperative Outcome.

Eine 81-jährige Patientin ist die Treppe hinuntergestürzt und hat sich eine zweitgradig offene Unterschenkelfraktur hinzugezogen. Sie ist für ihr Alter äusserst rüstig und wünscht, so rasch wie möglich wieder nach Hause gehen zu können. Welches wäre der nächste sinnvolle Schritt?

Unmittelbar definitive Versorgung durch den traumatologischen Notfalldienst, um eine rasche postoperative Mobilisation zu gewährleisten.

Fixateur-externe, Fotodokumentation des Weichtteildefektes und Anlage eines Feuchtverbandes, am Folgetag Planung im orthoplastischen Team, dann definitive Frakturversorgung ggf. mit Weichteilrekonstruktion.

Sofortiger Verschluß der Weichteile und Frakturversorgung im Anschluss rein traumatologisch, Weichteilprobleme können problemlos zu einem späteren Zeitpunkt plastisch-rekonstruktiv versorgt werden.

Bei rüstigen, gesunden traumatologischen PatientInnen braucht es keine interdisziplinäre Beurteilung. Dies verzögert und verkompliziert das weitere Vorgehen nur unnötig und verursacht zusätzliche Kosten.

Bei über 80-jährigen PatientInnen sollten keine aufwändigen Rekonstruktionen mehr durchgeführt werden, da diese überproportional mit schwerwiegenden Komplikationen vergesellschaftet sind.

Ein 89-jähriger Patient wird 6 Wochen nach Versorgung einer Außenknöchel-fraktur aus einem externen Krankenhaus zugewiesen. Seit drei Wochen zeigt sich im distalen Anteil des Zuganges ein Porus, aus welchem sich gelegentlich wenig klare Flüssigkeit entleert. Welches ist das ideale weitere Vorgehen?

Diese Wundheilungsstörung ist nicht weiter schlimm und ein Unterdruckverband wird das Problem mit grosser Wahrscheinlichkeit beheben.

Da der Patient nie Fieber hatte und die Sekretion immer klar war ist ein infektiöses Geschehen sehr unwahrscheinlich. Die Wunde kann in Lokalanästhesie verschlossen werden.

Es muss von einer Fraktur-assoziierten Infektion ausgegangen werden, eine orthoplastische Beurteilung ist zwingend, um interdisziplinär das weitere Behandlungsregime festzulegen.

Da nur ein begrenzter Defekt vorliegt, braucht es zunächst keine spezifische Therapie, die Wunde ist regelmässig auszuduschen, zu verbinden und der Patient in 6 Wochen nachzukontrollieren.

Eine angiologische Beurteilung ist unnötig, da diese häufig keine therapeutische Konsequenz nach sich zieht und die Fraktur bereits versorgt ist.
